# FAMILY HISTORY IS ASSOCIATED WITH THE PRESENCE OF DYSLIPIDEMIA IN PRE-SCHOOL CHILDREN

**DOI:** 10.1590/1984-0462/;2019;37;1;00005

**Published:** 2018-07-26

**Authors:** Mariana de Santis Filgueiras, Sarah Aparecida Vieira, Andréia Queiroz Ribeiro, Juliana Farias de Novaes

**Affiliations:** a Universidade Federal de Viçosa, Viçosa, MG, Brasil.

**Keywords:** Child, Parents, Dyslipidemia, Hypertriglyceridemia, Cardiovascular diseases, Criança, Pais, Dislipidemias, Hipertrigliceridemia, Doenças cardiovasculares

## Abstract

**Objective:**

To evaluate the association between family history and the presence of dyslipidemia in children.

**Methods:**

A cross-sectional study with 257 children aged 4 to 7 years old from Viçosa, Minas Gerais, Southeast Brazil. Nutritional status and lipid profile (total cholesterol, cholesterol fractions, and triglyceride) assessments and an active search for a family history of dyslipidemia in parents were carried out. Pearson’s chi-square test was used to identify associations, and Student’s t-test was used to compare means. A Poisson regression analysis was performed to assess the independent association between family history and the presence of dyslipidemia in children. A significance level of 5% was adopted.

**Results:**

Children of parents with dyslipidemia had higher serum concentrations of total cholesterol and triglycerides. In a regression analysis after adjustments, the presence of dyslipidemia in the father or in the mother (OR: 2.43; 95%CI 1.12-5.27), as well as the presence of dyslipidemia in both the father and the mother (OR: 5.62; 95%CI 2.27-13.92) were associated with hypertriglyceridemia in children. Children of parents with dyslipidemia had a higher prevalence of elevated low-density lipoproteins (LDL-c) (OR: 1.52; 95%CI 1.18-1.97).

**Conclusions:**

An investigation of the family history of dyslipidemia should be made as part of the protocol to verify the presence of hypertriglyceridemia and dyslipidemia in children.

## RESUMO

**Objetivo:**

Avaliar a associação entre história familiar e presença de dislipidemias em crianças.

**Métodos:**

Estudo transversal com 257 crianças de 4 a 7 anos de idade do município de Viçosa, Minas Gerais. Foram realizadas avaliações do estado nutricional e do perfil lipídico (colesterol total e frações e triglicerídeos), além do registro de história de dislipidemia dos pais. Para análise estatística, foi utilizado o teste do qui-quadrado de Pearson para identificar associações e teste *t* de Student para comparação de médias. Foi realizada análise de regressão de Poisson para avaliar a associação independente entre história familiar e a presença de dislipidemia em crianças, sendo adotado o nível de significância estatística de 5%.

**Resultados:**

Crianças com pais dislipidêmicos apresentaram maiores concentrações séricas de colesterol total e triglicerídeos. Na análise de regressão após ajuste, a presença de dislipidemia no pai ou na mãe (RP: 2,43; IC95% 1,12-5,27) bem como a presença de dislipidemia no pai e na mãe (RP: 5,62; IC95% 2,27-13,92) estiveram associadas à hipertrigliceridemia nas crianças. Crianças com pais e mães dislipidêmicos apresentaram maior prevalência de lipoproteína de baixa densidade (LDL-c) elevada (RP: 1,52; IC95% 1,18-1,97).

**Conclusões:**

A investigação da história familiar de dislipidemia deve fazer parte de protocolos para verificar a presença de hipertrigliceridemia e dislipidemias na infância.

## INTRODUCTION

Dyslipidemia occurs when lipoproteins are metabolically altered such that high serum concentrations of total cholesterol (TC), low density lipoproteins (LDL-c) and triglycerides (TG) are observed, as well as low serum concentrations of high density lipoproteins (HDL-c).^1^ Genetic factors, being overweight, sedentary behavior and poor nutrition may contribute to the onset of childhood dyslipidemia.^2^ ^,^ ^3^

The presence of dyslipidemia is the basis for the development of atherosclerosis, which can begin in childhood and extend into adolescence and adulthood.^4^ Therefore, the identification of children at risk for dyslipidemia is essential in the effort to reduce morbidity and mortality due to cardiometabolic diseases.^5^

Family history is one of the main factors that can detect dyslipidemia, as it is based on heredity, eating habits and the lifestyles shared by familes.^6^ Parents are actively involved in the formation of children’s eating habits and physical activity routines since they are the ones that provide food and set examples of eating habits and meal times. Additionally, they pass on their preferences for specific foods and brands.^7^

Considering the influence of family history on children’s health, knowing if dyslipidemia is present in the family history may contribute to more effective public health measures. Brazilian studies that deal with this subject are scarce, since, for the most part, family history of dyslipidemia is not an evaluated as a main variable. Thus, the objective of this study was to evaluate the association between family history and the presence of dyslipidemia in children.

## METHOD

The present research presents a cross-sectional study of children belonging to a retrospective birth cohort at the only maternity hospital in Viçosa, Minas Gerais. The children received medical supervision from the Lactation Support Program (*Programa de Apoio à Lactação* - PROLAC) in the first year of their life and were reassessed between the ages of 4 to 7 years old. PROLAC is a program of the Universidade Federal de Viçosa (UFV) in partnership with the municipality’s Human Milk Bank (*Banco de Leite Humano* - BLH).

This age group was decided upon due to the fact that their growth rate was practically constant between the end of their third year of life and the beginning of puberty.^8^

**Figure 1:**
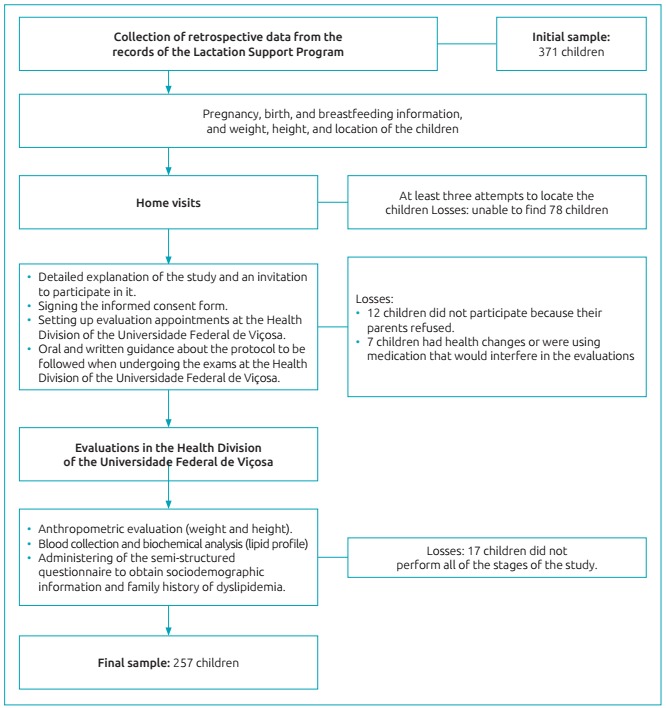
Schematic representation of the data collection and the study sample.

Based on information collected from PROLAC care records, and considering the inclusion criteria of the study - identification data that revealed the children’s location, if they had a full-term birth, appropriate weight, and a date of birth compatible with the ages between 4 and 7 years old at the time of the study - 371 children were eligible for this study. Of the eligible children, 78 were not located (change of address) after at least 3 attempts to locate the children through home visits, 12 were not authorized by their parents to participate, 17 did not complete all stages of the study and 7 had health problems that prevented them from participating. Thus, the final sample of the present study was 257 children. A schematic representation of the data collection and sample losses are shown in Figure 1.

Based on the means and standard deviations of children of mothers with dyslipidemia (178.0 ± 37.6 mg/dL) and mothers without dyslipidemia (165.2 ± 23.3 mg/dL), the sample had a power of 98.9% for a confidence level of 95%. The analysis was performed using the Open Epi Online Program.

The gender and age of the children were obtained through a semi-structured socioeconomic and demographic questionnaire, which was then used as an adjustment in the Poisson regression analysis. Gender was dichotomously categorized and age was assessed as a continuous variable (months).

To evaluate nutritional status, the following items were measured: weight, from an electronic digital scale with a maximum capacity of 150 kg and a sensitivity of 50 g; height, using a vertical stadiometer attached to the wall, with a range of 2m, divided into centimeters and subdivided into millimeters according to standards recommended by Jelliffe.^9^ WHO AnthroPlus software (World Health Organization, Geneva, Switzerland)^10^ was used to obtain body mass index by age (BMI/A) in a Z score, and to diagnose the nutritional status of the children, according to growth curves from the World Health Organization (WHO), and adopted by the Ministry of Health.^11^ ^,^ ^12^ For the purpose of analysis, overweight and obese children were classified as being overweight.

After the children had fasted for 12-hours, their blood samples were collected for an analysis of the following biochemical parameters: TC, LDL-c, HDL-c, and TG.

The analyses were performed from the blood serum after the material was centrifuged in the Centrifuge model 206BL Excelsa (FANEM, São Paulo, Brazil) for 10 min at 3,500 rpm. TC, HDL-c and TG were measured according to the enzymatic colorimetric method, and automated using Cobas Mira Plus equipment (Roche Corporation, Montclair, USA). LDL-c serum concentrations were calculated using the Friedwald formula, since no TG value was greater than 400 mg/dL.^13^ In addition, the ratios between TC/HDL-c, LDL-c / HDL-c and TG / HDL-c were calculated. These ratios accurately predict the presence of subclinical atherosclerosis and coronary disease in children and adults.^14^ ^,^ ^15^

The lipid profile was classified according to the I Guidelines for the Prevention of Atherosclerosis in Childhood and Adolescence from the Brazilian Society of Cardiology (*I Diretriz de Prevenção da Aterosclerose na Infância e na Adolescência da Sociedade Brasileira de Cardiologia*).^15^ A change in at least one lipid profile marker was considered sufficient to classify dyslipidemia.^16^

The family history of dyslipidemia was self-reported through a semi-structured questionnaire filled-out by parents. There was considered to be a family history of dyslipidemia when the father or mother also had dyslipidemia. 

Statistical analyzes were performed using the Social Package Statistical Science program (SPSS) for Windows version 20 (IBM Corporation, Chicago, USA). The Kolmogorov Smirnov normality test was applied, and a descriptive analysis of the data was carried out using frequency, central tendency and dispersion measurements. Pearson’s chi square test was performed to identify associations between the frequency of changes in the lipid profile and family history of dyslipidemia. The Student’s t test was used to compare the mean values ​​of the lipid profile markers between the two independent groups (children with and without a family history of dyslipidemia). Analysis of variance (ANOVA), with Tukey’s post hoc test, was used for comparing the three groups (no family history; father or mother has dyslipidemia; and father and mother both have dyslipidemia).

Poisson regression models with robust variance were used to evaluate the association of family history of dyslipidemia (independent variable) with hypercholesterolemia, low ­HDL-c, high LDL-c, and hypertriglyceridemia in children (dependent variables). The analysis was adjusted for gender, age and nutritional status (normal weight and overweight individuals). The suitability of the regression models was assessed by the Hosmer Lemeshow test (Goodness of fit test). For all of the tests performed, the level of statistical significance adopted was 5%.

This study was approved by the UFV Human Research Ethics Committee (nº 094/2011), and the children were included only after their parents or guardian signed an informed consent form.

## RESULTS

A total of 257 children, with a mean age of 71.5 ± 12.5 months (data not shown) were evaluated. The majority of them were male (55.3%) and had elevated TC (75.1%) and LDL-c (56.4%). The prevalence of family history of dyslipidemia was 22.6% -- 11.8% in mothers and 17.5% in fathers (data not shown). The family history of dyslipidemia was associated with the presence of hypertriglyceridemia in children (Table 1).

**Table 1:** Changes in the lipid profile according to family history of dyslipidemia in children.

	Total	Family history of dyslipidemia		p-value
		Yes	No	
	n (%)	n (%)	n (%)	
↑TC (≥150 mg/dL)				
Yes	193 (75.1)	47 (24.4)	146 (75.6)	0.235
No	64 (24.9)	11 (17.2)	53 (82.8)	
↓HDL-c (<45 mg/dL)				
Yes	87 (33.9)	25 (28.7)	62 (71.3)	0.091
No	170 (66.1)	33 (19.4)	137 (80.6)	
↑LDL-c (≥100 mg/dL)				
Yes	145 (56.4)	35 (24.1)	110 (75.9)	0.493
No	112 (43.6)	24 (20.5)	88 (79.5)	
↑TG (≥100 mg/dL)				
Yes	29 (11.3)	15 (51.7)	14 (48.3)	<0.001*
No	228 (88.7)	44 (19.3)	184 (80.7)

TC: total cholesterol; HDL-c: high density lipoprotein; LDL-c: low density lipoprotein; TG: triglyceride; Pearson’s chi square test; *p<0.05.

We observed that children with a family history of dyslipidemia had higher serum concentrations of TC (p=0.016), TG (p<0.001), and higher TC / HDL-c (p=0.010) and TG / HDL-c (p=0.002) ratios (Table 2).

**Table 2:** Lipid profile markers according to family history of dyslipidemia in children.

	Total	Family history of dyslipidemia		p-value
	(n=257)	Yes (n=59)	No (n=198)	
	Mean±SD	Mean±SD	Mean±SD	
TC (mg/dL)	166.8±26.1	174.0±31.1	164.6±24.2	0.016*
HDL-c (mg/dL)	50.8±11.5	49.7±12.6	51.1±11.2	0.445
LDL-c (mg/dL)	103.1±23.1	108.1±26.6	101.6±21.8	0.059
TG (mg/dL)	64.9±29.6	80.8±37.6	60.2±25.1	<0.001*
CT/HDL-c	3.4±0.8	3.6±0.9	3.3±0.8	0.010*
LDL-c/HDL-c	2.1±0.7	2.3±0.7	2.1±0.7	0.051
TG/HDL-c	1.4±0.9	1.8±1.1	1.3±0.7	0.002*

SD: standard deviation; TC: total cholesterol; HDL-c: high density lipoprotein; LDL-c: low density lipoprotein; TG: triglyceride; Student’s t test; *p<0.05.

**Figure 2:**
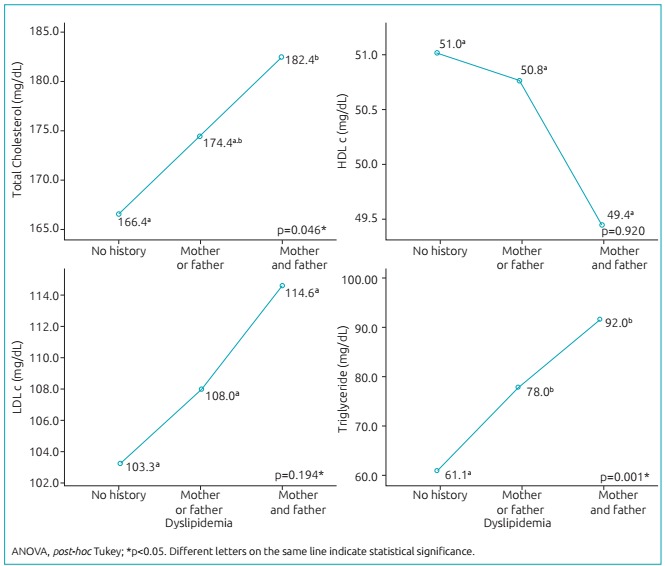
Lipid profile markers in accordance with family history of dyslipidemia.

Children of parents with dyslipidemia had higher serum concentrations of TC and TG compared to those without a family history of dyslipidemia (Figure 2). In the regression analysis, we observed that the presence of dyslipidemia in both the father and the mother increased the prevalence of elevated LDL-c in children (PR: 1.52; 95%CI 1.18-1.97; p=0.001). The presence of dyslipidemia in at least one of the parents increased the prevalence of hypertriglyceridemia in children (PR: 2.43; 95%CI 1.12-5.27; p=0.020); and was even greater when both parents had it (PR: 5.62; 95%CI 2.27-13.92; p<0.001) (Table 3).

**Table 3:** Crude and adjusted prevalence ratios of changes in lipid profile according to family history of dyslipidemia in children.

	Family history of dyslipidemia		
	No history	Mother or father	Mother and father
↑TC (≥150 mg/dL)			
PR (95%CI) crude	1.00	1.09 (0.93-1.27)	1.16 (0.90-1.48)
p-value	-	0.29	0.25
PR (95%CI) adjusted	1.00	1.10 (0.95-1.29)	1.17 (0.91-1.50)
p-value	-	0.21	0.21
↓HDL-c (<45 mg/dL)			
PR (95%CI) crude	1.00	1.27 (0.82-1.96)	1.42 (0.66-3.06)
p-value	-	0.29	0.37
PR (95%CI) adjusted	1.00	1.20 (0.77-1.88)	1.42 (0.69-2.93)
p-value	-	0.41	0.35
↑LDL-c (≥100 mg/dL)			
PR (95%CI) crude	1.00	1.04 (0.79-1.37)	1.53 (1.17-2.00)
p-value	-	0.78	<0.01
PR (95%CI) adjusted	1.00	1.03 (0.78-1.37)	1.52 (1.18-1.97)
p-value	-	0.81	<0.01
↑TG (≥100 mg/dL)			
PR (95%CI) crude	1.00	2.58 (1.18-5.63)	5.47 (2.23-13.45)
p-value	-	0.02*	<0.001
PR (95%CI) adjusted	1.00	2.43 (1.12-5.27)	5.62 (2.27-13.92)
p-value	-	0.02*	<0.001

Adjusted for age, gender, and nutritional status; TC: total cholesterol; HDL-c: high density lipoprotein; LDL-c: low density lipoprotein; TG: triglyceride; PR: prevalence ratio; 95%CI: confidence interval of 95%; Hosmer-Lemeshow test (*Goodness of fit test*); p>0.05.

## DISCUSSION

This study demonstrated that a family history of dyslipidemia was associated with the worse lipid profile in children, with an emphasis on the presence of hypertriglyceridemia. The presence of dyslipidemia in both the father and the mother increased the prevalence of elevated LDL-c, regardless of gender, age and nutritional status.

Many studies have shown the importance of the family history of dyslipidemia for the development of various cardiometabolic alterations,^17^ ^,^ ^18^ however, the present study is one of the few that analyzes Brazilian children. Similar to the results found in the present study, a family history of dyslipidemia was a risk factor for its development in Chinese children.^19^ It is important to consider that the high prevalence of dyslipidemia in children may be associated with genetic and inappropriate lifestyle factors shared between parents and children, such as food consumption and amount of physical activity.^20^ ^,^ ^21^ ^,^ ^22^

Some studies with other populations have shown the importance of performing screenings to identify a family history of dyslipidemia in children.^23^ ^,^ ^24^ ^,^ ^25^ It is known that familial hypercholesterolemia is a common genetic cause of premature coronary disease and is an autosomal dominant disorder present even in individuals with a healthy lifestyle. A diagnosis of familial hypercholesterolemia is based on phenotypic criteria, involving high serum concentrations of LDL-c, plus a family history of elevated LDL-c, premature coronary disease and/or genetic diagnoses.^4^ However, an under-diagnosis of familial hypercholesterolemia in children is observed, which points to the need to implement regular screening strategies.^26^

It is worth mentioning that, at a pre-school age, parents have a greater influence over eating behaviors and lifestyle.^27^ ^,^ ^28^ Considering the high prevalence of lipid profile alterations in this sample, there is a need for public policies that stimulate family involvement in the adoption of healthy living habits.

We observed a high prevalence of high serum concentrations of TC (75.1%) and LDL-c (56.4%) in children. Other studies have reported similar results, such as the one performed in Diamantina, Minas Gerais, which demonstrated low serum concentrations of HDL-c (81.7%) and high serum concentrations of TC (49.3%), LDL-c (30.4%), and TG (2.6%) in pre-school children.^3^ In the state of Pernambuco, 24 and 6% of children and adolescents, respectively, had borderline and elevated serum concentrations of TC, and around 15 and 10% of the sample had borderline and elevated LDL-c serum concentrations.^29^ These results indicate the need to establish public policies that stimulate behavioral change in children, combating unhealthy eating habits and preventing the onset of cardiometabolic diseases.

Excess LDL-c particles cause atherosclerosis, a chronic inflammatory disease that occurs in response to endothelial aggression, and includes the innermost layer of medium- and large-caliber arteries.^30^ LDL-c is oxidized and accumulates in the artery wall, stimulating the formation of leukocyte adhesion molecules. In turn, these molecules attract lymphocytes and monocytes into the arterial wall. Monocytes differentiate into macrophages, which pick up oxidized LDL-c, forming the foam cells. These cells stimulate the secretion of cytokines and proteolytic enzymes, causing the destruction of endothelial tissue.^31^

Hypertriglyceridemia results from the accumulation of chylomicrons and/or very low-density lipoproteins (VLDL-c), because of the reduction of TG hydrolysis of these lipoproteins by lipoprotein lipase or by the increase in the synthesis of VLDL-c. Depending on the origin of the TG - whether it is exogenous (chylomicron) or endogenous (VLDL-c) - changes in dietary habits are required, which include a reduction in the intake of fats and/or carbohydrates, and an increase in physical activity.^1^

A family history of dyslipidemia was associated with higher TC/HDL-c and TG/HDL-c ratios. Studies evaluating this association are scarce. However, there is evidence in the literature of the association of these indices with higher concentrations of C-reactive protein (us-CRP),^32^ ^,^ ^33^ central obesity, insulin resistance, metabolic syndrome, and thickness of the inner and middle layer of the carotid artery in children.^33^

One of the limitations of this study was that the information regarding family history of dyslipidemia was self-reported. On the other hand, many studies have demonstrated that the self-reporting of diseases is valid and can be used in triage and epidemiological studies.^34^ ^,^ ^35^ Another limitation is the study’s cross-sectional design, which makes it impossible to infer a cause and effect relationship. Therefore, further studies are needed to assess how family history can determine the risk for dyslipidemia in children. This interaction is multifactorial, encompassing cultural, genetic and environmental aspects.^36^

This study allowed us to consider the importance of family history of dyslipidemia as a way to screen for the risks for hypertriglyceridemia and elevated LDL-c in children. Family involvement can be a way to prevent the onset of these changes in childhood and can even control these diseases that are already present in the parents.
